# Production, review, and impact of technical quality control guidelines in a national context

**DOI:** 10.1120/jacmp.v17i6.6422

**Published:** 2016-11-08

**Authors:** Michelle K. Nielsen, Kyle E. Malkoske, Erika Brown, Kevin Diamond, Normand Frenière, John Grant, Natalie Pomerleau‐Dalcourt, Jason Schella, L. John Schreiner, Laurent Tantôt, J. Eduardo Villarreal‐Barajas, Jean‐Pierre Bissonnette

**Affiliations:** ^1^ Department of Medical Physics Mississauga Halton/Central West Regional Cancer Program, Trillium Health Partners Mississauga ON Canada; ^2^ Radiation Treatment Program Simcoe Muskoka Regional Cancer Program, Royal Victoria Regional Health Centre Barrie ON Canada; ^3^ Canadian Partnership for Quality Radiotherapy Red Deer AB Canada; ^4^ School of Interdisciplinary Science McMaster University Hamilton ON; ^5^ Department of Medical Physics Juravinski Cancer Centre Hamilton Ontario Canada; ^6^ Département de radio‐oncologie Centre intégré universitaire de santé et de services sociaux de la Mauricie‐et‐du‐Centre‐du‐Québec, Centre hospitalier affilié universitaire régional Trois‐Rivières QC Canada; ^7^ Department of Radiation Oncology Dalhousie University Halifax NS Canada; ^8^ Cape Breton Cancer Centre Nova Scotia Health Authority Sydney NS Canada; ^9^ Département de Physique Médicale Centre d'oncologie Dr. Léon‐Richard, Réseau de Santé Vitalité Moncton NB Canada; ^10^ Medical Physics Team QEII Health Sciences Centre, Nova Scotia Health Authority Halifax NS Canada; ^11^ Medical Physics Department Cancer Centre of Southeastern Ontario/ Kingston General Hospital Kingston ON Canada; ^12^ Département de Radio‐Oncologie Centre intégré universitaire de santé et de services sociaux de l'Est‐de‐l’Île‐de‐Montréal – Hôpital Maisonneuve‐Rosemont Montréal QC Canada; ^13^ Department of Oncology University of Calgary Calgary AB Canada; ^14^ Department of Medical Physics Tom Baker Cancer Centre Calgary AB Canada; ^15^ Department of Radiation Oncology University of Toronto Toronto ON Canada; ^16^ Department of Medical Physics Princess Margaret Cancer Centre Toronto ON Canada

**Keywords:** quality assurance, quality control, radiotherapy, workload

## Abstract

A close partnership between the Canadian Partnership for Quality Radiotherapy (CPQR) and the Canadian Organization of Medical Physicist's (COMP) Quality Assurance and Radiation Safety Advisory Committee (QARSAC) has resulted in the development of a suite of Technical Quality Control (TQC) guidelines for radiation treatment equipment; they outline specific performance objectives and criteria that equipment should meet in order to assure an acceptable level of radiation treatment quality. The adopted framework for the development and maintenance of the TQCs ensures the guidelines incorporate input from the medical physics community during development, measures the workload required to perform the QC tests outlined in each TQC, and remain relevant (i.e., “living documents”) through subsequent planned reviews and updates. The framework includes consolidation of existing guidelines and/or literature by expert reviewers, structured stages of public review, external field‐testing, and ratification by COMP. This TQC development framework is a cross‐country initiative that allows for rapid development of robust, community‐driven living guideline documents that are owned by the community and reviewed to keep relevant in a rapidly evolving technical environment. Community engagement and uptake survey data shows 70% of Canadian centers are part of this process and that the data in the guideline documents reflect, and are influencing, the way Canadian radiation treatment centers run their technical quality control programs. For a medium‐sized center comprising six linear accelerators and a comprehensive brachytherapy program, we evaluate the physics workload to 1.5 full‐time equivalent physicists per year to complete all QC tests listed in this suite.

PACS number(s): 87.55.Qr, 87.56.Fc, 87.56.‐v

## I. INTRODUCTION

Radiation treatment is indicated for approximately 52% of all incident cases of cancer at some point during the management of the disease.^(1)^ A challenge that is general to all radiation treatment centers is the delivery of safe, state‐of‐the‐art treatment to all patients in a fiscally‐constrained and regulatory‐rich environment where commercial platforms evolve at a rapid pace. This context also includes an interdisciplinary team of radiation oncologists, medical physicists, medical radiation therapists, and other professions who recognize that treatment is a complicated process that involves multiple handovers and input from all disciplines. Finally, in Canada, radiation treatment is delivered in 47 facilities; in a socialized medicine context, it is desirable to offer all Canadian patients treatment of equivalent quality, independent of the elected center.

In an effort to drive this quality improvement agenda at the national level, the Canadian Partnership for Quality Radiotherapy (CPQR) was formed as an alliance of the three key national professional organizations involved in radiation treatment in Canada: the Canadian Association of Radiation Oncology (CARO), the Canadian Organization of Medical Physicists (COMP), and the Canadian Association of Medical Radiation Technologists (CAMRT). The federal government provides support through the Canadian Partnership Against Cancer (CPAC), a national resource for advancing cancer prevention and treatment. The mandate of the CPQR is to support the universal availability of high‐quality and safe radiotherapy for all Canadians through system performance improvement and the development of consensus‐based guidelines and indicators to aid in radiation treatment program development and evaluation. CPQR does this through the development of consensus‐based guidelines and quality indicators for radiation treatment program development and evaluation, and through partnership on initiatives aimed at driving compliance with such indicators, such as the partnership with Accreditation Canada on the integration of a radiation treatment module into its standards accreditation process.^2^ *The Quality Assurance Guidelines for Canadian Radiation Treatment Programs* ^(2)^ establishes a benchmark for achievement in the areas of programmatic quality and safety and details key quality indicators essential to programmatic assessment.

In this manuscript, we present the suite of Technical Quality Control (TQC) documents. Each TQC document details relevant QC tests to be performed, specifying the recommended test frequency and tolerances for an acceptable level of equipment performance. The development of each individual TQC guideline is spearheaded by an expert team. However, a unique feature of our approach is that input from the medical physics and radiation therapy communities is acquired at various steps during the development process, with emphasis on the external validation of each QC test suite prior to endorsement. The long‐term objectives are to close the loop between tolerances defined by experts and QC data that are actually acquired by staff physicists in their respective centers and to improve compliance to guidelines by involving a large contingent of physicists at various steps of the production. This manuscript explains the production process for this suite of documents and reports on their acceptability and impact in the Canadian clinical context.

## II. MATERIALS AND METHODS

### 1. Justification: a stakeholder analysis

The TQC suite's initial objective was to overhaul an existing set of QC “standards” that the Canadian Association of Provincial Cancer Agencies (CAPCA) produced (henceforth referred to as the “CAPCA standards”^(3)^). The CAPCA standards were regarded by regulators as absolutes to which clinics had to comply despite their lack of input. Despite this pressure, the CAPCA standards fell quickly into disuse for a number of reasons.^(4)^ No Canadian radiation treatment programs exceeded a compliance level of 90% to any of the tests. Several QC tests had degrees of noncompliance of up to 70%‐80%.^(4)^ This finding reflects competing demands on machine access between additional QC work and clinical throughput.^(5)^ To formally identify and address other barriers to the adoption of national quality control guidelines, we used change‐management techniques, starting with a thorough stakeholder analysis.^(6)^

One such barrier is that the preparation, revision, and publication of QC guidelines by any national organization of medical physicists take a long time. This is due, in part, to the voluntary nature of the process. In the absence of incentives, other activities naturally took precedence over voluntary tasks. Reviewing long, in‐depth documents and processing them through complicated hierarchy involving multiple organizations further slowed down the process. An unfortunate consequence is that publication of QC guidelines lags behind the introduction of new technology by a few years; therefore, at the rapid pace technology is evolving in our field, QC guidelines can become rapidly out of date.

The second criticism is that the rationale and urgency to perform some of the QC tests is not well understood. Basing QC guidance on formal risk assessment tools, such as failure mode and effects analysis,^(7)^ would clarify the justification for this additional work if it is done well, but in‐depth risk assessment remains daunting for individual clinics. Another facet of this criticism is that QC test frequencies and tolerances found in guideline documents are rarely obtained from a formal analysis of QC test results using statistical process control (SPC) and sampling theory.^8^, ^9^, ^10^ Therefore, previous guidance provided by experts may be unjustified or have gaps.

The third criticism was that the CAPCA standards were promoted as standards, not realizing the full repercussions of this word; therefore, the documents were treated as absolutes by regulatory agencies and hospital administrators, and the latter often required full compliance to the test suite without providing adequate resources or access on the equipment to be tested. Given that the end users had reason to question the credibility of the QC guidance documents, their questions changed to concerns when the Canadian Nuclear Safety Commission (CNSC) began to ask for records of compliance to the potentially out‐of‐date CAPCA standards.^(3)^

In order for this new set of TQC guidelines to be successful, an action plan was designed, based on change‐management theory, to bring resolutions to these problems.^(11)^ In order to enhance buy‐in and raise compliance, a clear vision was provided for a participative approach that motivated physicists across the country to produce, comment on, and endorse these new guidelines. Also, resources were allocated to reward volunteer participants by subsidizing participation in relevant meetings, such as the Canadian Winter School, where their contributions were immediately recognized by their peers.^(11)^

**Figure 1:**
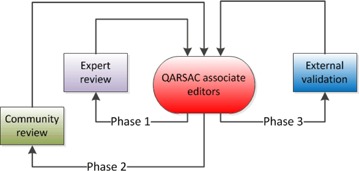
Revision cycles of the Technical Quality Control document review process.

This process, shown in Fig. 1, has introduced a number of novel ideas, including the concept of “living document”: every few years, the original expert reviewer team is asked to revise their document, accounting for new literature, feedback from users, technology advancement, or QC data that would enable a revision of test tolerances and frequencies according to SPC or sampling theory. COMP would proceed subsequently to officially endorse the document and recognize the authors. Infrastructure and administrative assistance is provided by CPQR to encourage experts to stick to their timelines.

To improve the compliance of Canadian medical physicists to the new suite of guidelines, an external validation process was built into the development framework. This process includes the identification of Canadian radiation treatment centers to field‐test each TQC guideline prior to endorsement. Centers were chosen to represent a variety of sizes, clinical orientations (academic or community clinics), equipment manufacturers, and regions. Each center was asked to perform the QC test schedule in its entirety within a given time frame. Participating centers were also asked to document their perception of the value of each test, failure modes associated to each test, to identify the hazard mitigated by the test, and to measure the time, in man‐hours, required to complete the test suite. The original expert reviewer team was subsequently asked to respond and update the guideline using the feedback thus provided.

The development framework also addresses the issue of misinterpretation of QC guidance documents by regulators by actively involving them in TQC documents that are clearly within their scope. For example, the CNSC is interested in testing of safety systems present in radiation treatment centers. Therefore, they were invited to actively participate in the drafting of a new safety systems TQC guideline to ensure that the reference document used by physicists is fully compliant with regulations, and that the regulatory body has an understanding of the application and compliance requirements with the TQC. Another factor contributing to the uptake and acceptance of the TQC guidelines by radiation treatment centers generally, and the medical physics community specifically, is that the new suite of documents was clearly labeled as “guidelines” rather than “standards,” thereby softening the official stance.

With infrastructure and resources in place, it is thought that the revised guideline production process can be nimble and, with active participation of several Canadian radiation treatment centers, the validation workload can be shared across a large number of individuals, buy‐in can be enhanced, and gaps in the suite of guidelines documents can be identified quickly.

### 2. Document structure

The suite was designed using a common structure, as outlined by Dunscombe et al.^(12)^ Briefly, the format and introductory text of each document was made uniform; the authors of an individual TQC would thus only have to write a system description and focus their work on the actual list of QC tests, including their test frequencies and tolerances. This has kept terminology clear and succinct. The structure therefore facilitates and accelerates drafting and reviewing of a large amount of data with the emphasis on performance measures and indicators, as opposed to editing and wordsmithing. Each equipment‐specific TQC guideline contains concise descriptions of radiotherapy equipment and performance objectives to ensure geometric or dosimetric integrity. The safety systems TQC guidelines contain functional tests of the facility's auxiliary safety systems associated with radiotherapy equipment (e.g., door interlock, last‐person‐out circuit). They are separated from the equipment‐specific guidelines and consolidated to ensure a consistent approach to safety and focus the attention of the regulators. Separating the safety system tests for a given piece of equipment also allows the overall guideline suite to more easily be adapted to the specific regulatory framework of other countries and jurisdictions.

### 3. Document generation and review framework

The process for developing a TQC guideline involves several steps and engages many stakeholders. COMP's QARSAC committee manages suggestions for new TQC guidelines and their development. This national process (Fig. 1) is intended to provide rapid, relevant and practical guidance for quality control of radiotherapy equipment.

#### 3.1 Phase 1: expert review

The generation of a guideline document for a specific technology has been driven by clinical practice and necessity. The volunteer expert reviewers are responsible for the guideline during this development stage (Phase 1); system description, initial quality control test list, tolerances, and frequencies are drafted from a review of available literature, including existing guidelines from professional associations, related to the pertinent equipment.

#### 3.2 Phase 2: community review

Once an initial draft by the expert reviewer team is prepared, the guideline is shepherded through a Phase 2 community review that consists of an online review and comment period of at least 30 days. Comments are accepted from the community at large to promote concise feedback and review of suggested testing methods and frequencies by a variety of interested parties in a multitude of clinical settings. The community comments are sent back to the expert reviewer team for incorporation of feedback, refinement, and validation.

#### 3.3 Phase 3: external validation

For Phase 3, the revised guideline documents were sent to selected clinics who were asked to beta‐test the proposed QC guideline and provide feedback, including usability and workload data. The number of clinics asked depended on the guideline being reviewed. To ensure a breadth of engagement and avoid biased guidelines, we ensured that diverse clinics, in terms of geography, size, academic vs. community orientation, and diversity of manufacturers were represented. Typically, this meant 3–5 clinics beta‐tested each guideline. We asked these clinics to complete all the tests included in the document they were testing and asked that they adhere to the test frequencies outlined in the document. Volunteer clinics were also asked that all tests, including the annual ones, be completed within a three‐month time frame. The information gathered in this process is standardized by the use of a report template issued to the external validation institutions. As stated for the community review, the results and comments are sent back to the initial expert reviewer team for incorporation into the guideline document. The final document is then endorsed by the COMP board, translated into French, and uploaded to the CPQR website (www.cpqr.ca).

### 4. Living documents: ongoing review

We plan to review each TQC guidance document every three years to keep the documents current and continuously refreshed, inputting new expert findings, risk assessments, and accumulated QC data, and to account for the pace of technological evolution. By retaining ownership of these “live” documents, COMP and CPQR can also rapidly address any issues raised by regulators or the community. As of this writing, two guidelines are undergoing ongoing review.

### 5. Measurement of impact

We surveyed physicists to assess the impact of each of the TQC guidelines. At the time of the survey distribution, nine TQC guidelines had been ratified and posted.^(13)^ The survey was distributed to the heads of physics at all of the 47 Canadian radiation treatment facilities, as well as to QARSAC members. Respondents were asked to complete one survey on behalf of their institution. The impact was assessed by querying whether institutions had made changes to their QC programs, rating their compliance, and gauging their guideline implementation plans. Geographical data were captured to ensure good representation from across the country. Surveys were offered in both English and French.

## III. RESULTS

**Figure 2:**
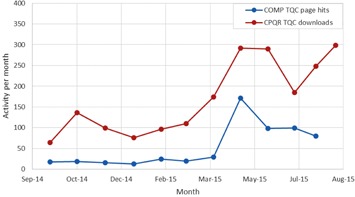
Technical Quality Control document cumulative webpage hits September 2014 to August 2015.

**Figure 3:**
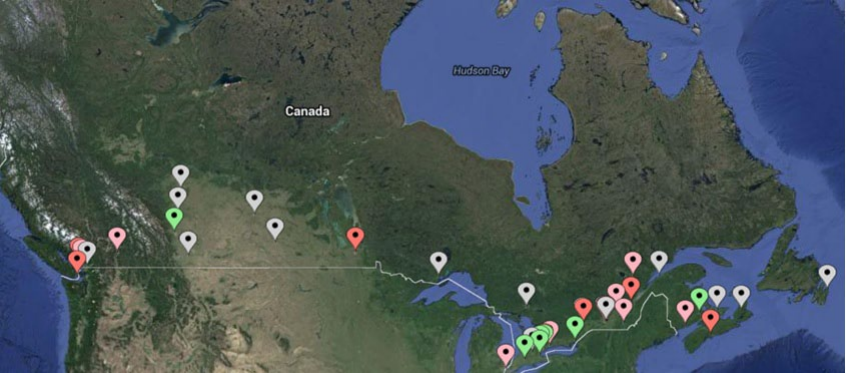
Canadian map showing all radiotherapy centers. Red and Pink markers represent large and small external validation centers, respectively. Green markers represent centers where expert reviewers are employed. White makers indicate centers that have yet to contribute.

The current state of the TQC guideline suite has nine finalized documents and 10 documents in some part of the review process, as shown in Table 1. The first nine documents took an average of 18 months (range: 15–24 months) to complete Phases 1 and 2; at that point they were posted as reference to the community on http://www.cpqr.ca/programs/technical‐quality‐control/. It took an additional 10 months to complete Phase 3 (external validation), translation into French, and ratification. Figure 2 shows that the documents have had impressive uptake in the community, as demonstrated by the over 2,000 downloads counted between September 2014 and August 2015. Table 2 shows that, with 25% of downloads originating from outside of Canada, there has been demonstrated international interest. Figure 3 shows that community engagement is high in all areas of review, with 70% of Canadian centers taking part in the guideline development/review process.

**Table 1:** List of documents available on http://www.cpqr.ca/programs/technical‐quality‐control/www.cpqr.ca. Accessed May 20, 2016

*Technical Quality Control Guideline*
*Community Review/ Expert Review (draft by expert reviewers)* CYB: CyberKnife
*External Validation (field/beta testing)*
RED: Reference Dosimetry
PDM: Patient‐Specific Dosimetric Measurements for Modulated Therapies
SST: Safety System Technical Quality Control
GKR: Gamma Knife Radiosurgery
RDM: Data Management Systems
TOM: Helical Tomotherapy
4DC: Four Dimensional Computed Tomography
*Edits & Translation*
CTS: Computed Tomography Simulators
VMA: Volumetric Modulated Arc Therapy (incorporation into MLA)
*Finalized/Ratified*
ACB: Accelerator Integrated Cone Beam Systems for Verification Imaging
LDR: Low Dose Rate Permanent Seed Brachytherapy
MLA: Medical Linear Accelerators and Multileaf Collimators
TPS: Treatment Planning Systems
HDR: Brachytherapy Remote Afterloaders
MDE: Major Dosimetry Equipment
CRS: Conventional Radiotherapy Simulators
KRM: Kilovoltage X‐ray Radiotherapy Machines
BRA: Brachytherapy Remote Afterloaders

**Table 2:** Summary of regional download data from www.cpqr.ca between September 2014 and August 2015

*Region*	*Number of Downloads*	*%*
Canada	1486	72.1%
Europe	204	9.9%
Asia	145	7.0%
Africa	99	4.8%
North America (excl. Canada)	64	3.1%
Australia/Oceania	54	2.6%
South America	8	0.4%

### 1. Case study: guideline for medical linear accelerator

We now present the production of the medical linear accelerator and multileaf collimator (MLA) TQC guideline as a case study to illustrate the production process, understanding that similar conclusions can be drafted from any other TQC guideline. Three physicists from Alberta and Ontario were assigned as expert reviewers. The initial draft was completed on Feb 17, 2012, and posted on the COMP website for 30 days of community review.

#### 1.1 Community review

During the community review phase, 76 comments were received from nine individuals, each from a different clinic. The expert reviewer team responded to each comment. Table 3 shows an example of the tools used to keep track of comments and their respective responses.

**Table 3:** Sample comments and responses from community review of the MLA TQC guideline. Test acronyms are indicated in parentheses

*Comment from Community*	*Response from Expert Reviewers*
Test DL7 (Room Radiation Monitors): We only have one radiation monitor in our treatment room. Should it be “Room radiation monitor” (no “s”)?	*Action taken:* Test descriptor was changed to ‘Radiation monitoring system’.
When looking at positioning accuracy, in particular the laser/crosshair daily QC, was any consideration given to the tolerance for IMRT set by TG142 of 1.5 mm?	*No action taken:* Given that CPQR states both a tolerance and action level (1 and 2 mm, respectively), we consider that the test is reasonably aligned with TG142.
It would appear that there are no open field profile or depth dose measurements in the annual tests but that the profile requirements for the monthly tests have been increased substantially. Is this your intent or is it an omission? TG142 does include annual tests for the change of flatness and symmetry from baseline for example. I am also not confident enough in our monthly profile measurement techniques to make significant beam adjustments based solely on these measurements and would thus continue to do annual measurements in a water tank.	*Action taken:* Test AL1 (Profile reproducibility) and AL2 (Depth dose reproducibility:) have been added to specifically describe water‐tank scanning or an acceptable surrogate on an annual basis. The monthly testing of PDD and profile is meant to be a simple point measurement check, whereas the annual test establishes complete and consistent curves.
Test ML16 (Light/radiation coincidence): To my mind, the tolerance and action levels are too large. It should be 1 and 2 mm.	*No action taken:* These limits were discussed extensively at the COMP Winter School and the consensus was that 2 and 3 mm were acceptable.

#### 1.2 External validation

The revised guideline was sent for external validation to centers in British Columbia and Québec on April 1, 2013. The last completed report was received on Feb 7, 2014. Some sample comments and responses are shown in Table 4. Workload measurements from the external validation centers revealed a variation of approximately 30% in the time taken to perform daily, monthly, and annual tests between the centers.

Following final edits and French translation, the MLA TQC was ratified by COMP and the finalized guideline was published online on Feb 28, 2015. This first version did not include any tests related to volumetric‐modulated arc therapy (VMAT), which had exploded in use while the MLA was being developed. QARSAC recognized the need to produce a TQC guideline for VMAT and build consensus towards its quality control. The VMAT TQC guideline has recently completed field‐testing at three centers in Canada, and is available at http://www.cpqr.ca/programs/technical‐quality‐control/. The TQC development process identified requirements for some additional monthly tests for VMAT (related to the ability of the linac to simultaneously control dose rate, gantry speed, and MLC positioning). These tests were recently merged into the existing MLA TQC guideline, resulting in a more comprehensive and up‐to‐date document for linear accelerator QC. It should be noted that, although the TQC guideline production can be long due to the extensive peer review process (approximately three years in the case of the MLA TQC), the documents are made publicly available throughout on www.cpqr.ca, with clear identification of the document's current status in the development process.

**Table 4:** Sample comments and responses from external validation of the MLA (Medical Linear Accelerators and MLC) TQC guideline. Test acronyms are defined in parentheses

*Comment from External Validation Center*	*Response from Expert Reviewers*
Test DL7 (Room radiation monitoring system): We believe that this system adds no benefit to security, nor quality, bearing in mind economic considerations, and should not be encouraged by the TQC guidelines. We suggest removing the test from TQC guideline. Similar comment received from a second institution during external validation.	*Action taken:* Review of federal regulations show no requirement for an in independent radiation room monitoring system for linear accelerators. The test has been removed from the TQC guideline.
Citing an example procedure may clarify the implementation of certain tests. For example, it was not exactly clear from the description how Test AL14 (Coincidence of axes of rotation) and Test AL15 (Coincidence of radiation and mechanical isocentre) differ. The names may be mismatched from the description and the technical details of implementation could be clarified.	*Action taken:* Test descriptions changed to clarify that *AL14 (*Coincidence of radiation and mechanical isocentres) refers to the independent congruence of the radiation and mechanical isocentres for collimator, gantry and couch rotation, whereas Test AL15 (Coincidence of axes of rotation) refers to the diameter of the sphere that encompasses the mechanical axes of rotation of the collimator, gantry and couch.
Test AL1 (Profile reproducibility): The test description note does not specify the depth of measurement for electron beams, while it does for photon beams. We suggest specifying for both or remove it for photons.	*Action taken:* Specification of depths removed.

### 2. External validation questionnaire data

Centers that field tested individual TQCs were surveyed using generic questions (Table 5). Of note, there were centers that asked for changes to the test description, failure mode and frequencies at this stage of guideline production. Also, there were discussions regarding descriptions of tests that led to descriptions being modified to be more specific and mitigated future confusion, as shown in Table 4.

There is overwhelming agreement (see Table 5) with the centers that took part in external validation testing, that the procedures and frequencies reflected current practices and technologies. There was also agreement that tests were appropriate and achievable.

Measured workload data seen in Table 6 are representative of nine documents validated by 18 centers. As noted in the section above, the time needed to complete all tests related to each TQC document varies widely. The data show that many hours are involved on completing quality assurance testing for each type of equipment and system. Assuming 260 operational days per year, and 7.5 worker‐hours per day, we present an equation that uses the average number of hours required to complete all QC tests to estimate the average FTE (physics full‐time equivalent) required to complete all of the QC tests compiled so far for a given cancer program,

$$FTE_{e}=\frac{[320L_{t}+25]+190K_{t}+10T_{t}+262D_{t}+159A_{t}+90S_{t}+98C_{t}}{1950}$$

where $FTE_{e}\;=$ the full‐time employee equivalent based on 1,950 hours worked per year; $L_{t}\;=$ total # of linear accelerators (with cone‐beam CT capabilities), $K_{t}\;=$ total # of kilovoltage treatment units, $T_{t}\;=$ total # of treatment planning systems, $D_{t}\;=$ total # of LDR systems, $A_{t}\;=$ total # of brachytherapy afterloaders, $S_{t}\;=$ total # of CT simulators, and $C_{t}\;=$ total # of conventional simulators.

The number of hours for major dosimetry equipment is assumed to be constant for all centers (i.e., 25 hrs, irrespective of the number of accelerators). We considered a medium‐sized center comprised of an external‐beam program of two CT simulators, six linacs, one kV treatment unit, and a brachytherapy program with two LDR and one HDR units with respective treatment planning systems. Using Eq. (1), we estimate the time to perform all quality control tests in a typical clinic (i.e., six linacs with CBCT, one orthovoltage unit, three treatment planning systems, one remote afterloader, two CT sims, and two LDR systems) would be 1.6 FTE or 2,842 hrs per year. A single linac equipped with CBCT thus requires 345 hrs of QC work per year, or 1.35 hrs per day, well below the 2.25–2.5 hrs per day per linac range estimated by Palta.^(5)^ To our knowledge, this is the first estimation of the actual resources for quality control activities based on actual data.

**Table 5:** Summary data and subset of questions from external review questionnaires (21 reports were submitted from 15 centers on 11 TQC documents), and percentage of tests (total number is 348 tests) that were commented for each section of the report

*Survey Question*	*% Positive Score*	*Comments*
Do any of the tests specified require changes to either tolerances or frequency?	15% (53/348)	Discussions or clarifications required for specific tests
Does your center have all the necessary resources to complete the tests outlined? Were all the tests performed?	83% (289/348)	Human Resources and/or equipment availability factors
Are the tests appropriate and achievable?	92% (320/348)	Related test specifications and vendor equipment differences
In your opinion, does the document address current practices and technologies?	91% (19/21)	2 centers did not answer

**Table 6:** Annual workload measurements, obtained from 18 external validation reports from centers on 9 TQC documents. Assumed test frequencies per year: daily 260, weekly 52, monthly 12, quarterly 4, annual 1, biennial one‐half

*Document*	*Responders*	*Average Hours*	*Minimum Hours*	*Maximum Hours*
ACB	2	123	118	129
LDR	2	262	220	304
MLA	2	197	164	230
TPS	2	10	10	11
BRA	2	159	94	224
MDE	2	25	15	35
CRS	2	98	70	125
KRM	2	190	158	223
CTS	2	90	78	102

### 3. Community impact survey data

**Figure 4:**
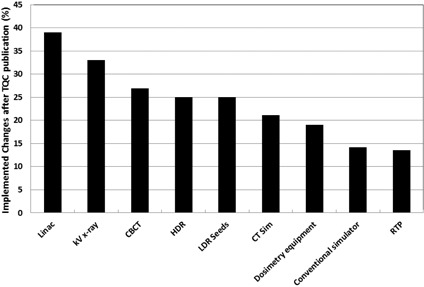
Percentage of respondents who made changes to their QA program following publication of the given TQC guideline. Respondents that did not possess the respective equipment were removed from the denominator.

Twenty‐eight of the surveyed centers provided a response (65%) across all regions of Canada. The impact of the TQCs, in terms of changes made to local QA programs, showed some dependence on the type of equipment (Fig. 4). The MLA TQC was most impactful, where nearly 40% of respondents indicated that they made changes to their in‐house QC test suite to better reflect the guideline. Overall, for the institutions that implemented changes, evaluation of their program at the time of publication revealed an average compliance of 52%. This was increased to 71% following the program modifications. Given the fact that only seven of the nine available guidelines were ratified at the time of the survey in 2014, we asked institutions about their implementation plans. Fifty‐three percent of all respondents indicated that they were planning to be fully compliant with the TQCs within the next 12 months. A further 25% were planning a partial implementation due to lack of human or equipment resources or to disagreement with some of the proposed tests. Finally, 12% of all respondents indicated that they were not planning further implementation, as they were already more than 95% compliant. This demonstrates that public posting of the documents allowed ample time for individual centers to examine and implement the guidelines prior to their formal ratification.

In order to compare uptake of the TQC guidelines with that of the predecessor CAPCA standards, we focused on linear accelerators with multileaf collimator and integrated kV imaging systems, which had previously been surveyed by Clark.^(4)^ Our survey showed that 70% of institutions reported compliance in the range of 81%–100% with the TQC guidelines. This is a marked improvement over Clark's findings,^(4)^ in which only half of the respondents indicated the same level of compliance with the CAPCA standards. This demonstrates the improved acceptance of the TQC guidelines by the community.

## IV. DISCUSSION

In a novel, Canada‐wide process, living technical quality control guidance documents are being produced with the hopes that they remain timely, relevant, and applicable. The process started in 2010 and since then 19 TQC guideline documents are within various stages of the production process. There are also two guideline documents that are approaching the end of their cycle and will commence the production of their second version shortly (ACB: accelerator integrated cone beam systems for verification imaging and LDR: low‐dose‐rate permanent seed brachytherapy); the overarching CPQR programmatic quality document^(2)^ is in its third revision. This demonstrates the commitment of Canadian centers to this initiative to continually improve quality and safety in our respective centers.

This production process has the unique ability to capture feedback on feasibility from the external validation centers; this dialogue helps address concerns from centers at an early stage of development. The documents have been peer‐reviewed extensively by a large number of clinical physicists and, unlike most QC guidance documents from professional bodies, have been thoroughly beta‐tested prior to ratification. In combination with timely translation into French, this approach therefore gives the TQC guidelines high impact and credibility. As well, the community‐led experts can communicate and collaborate with regulators and administrators so regulatory feedback can be used in the inception of the guideline, as opposed to later during the clinical application.

Our survey confirmed that, in a small number of centers, the challenges with limited human resources and equipment in performing the entire TQC test suites remain. It is hoped that Eq. (1) will help individual centers address the former challenge.

One criticism that our approach shares with most bodies providing QC guidance is that the test tolerance and frequencies are not systematically supported by quality management methodologies. Tools like statistical process control charts^(14)^ and failure modes and effects analysis^15^, ^16^ are becoming popular in our field to help generate and manage QC data. These data, in turn, are suitable for other quality management tools, such as acceptance sampling theory,^(10)^ to establish QC test tolerances and frequencies that are data‐driven as opposed to the consensual approaches used thus far.^(5)^ We suggest that future versions of the TQC guidelines poll QC data from many centers to revise QC test tolerances and frequencies in an effort to evolve into QC guidelines that are data‐driven and more efficient while maintaining the effectiveness of the QA program.

## V. CONCLUSIONS

CPQR and COMP have established a functional and sustainable process to produce and revise quality control documents that accounts for end‐user input in a meaningful way. The TQC suite is intended to remain current by cycling through this repeated peer‐review process. By providing necessary resources, we have shown that a large‐scale enterprise of this kind can be successful, relevant, and generalizable. By producing a body of work that stems from the community and reaches consensus, and by involving regulators as collaborators, we hope that endorsement and adoption will persist and transcend the national boundaries of Canada.

## ACKNOWLEDGMENTS

Authors would like to thank many people who were involved and were instrumental in the production of the TQC guideline suite. These include the COMP board of directors, as well as the CPQR Steering Committee members. As well, the authors would like to thank all the expert reviewers, community reviewers and all the centers that have participated in the TQC development process. The production of this manuscript has been made possible through a financial contribution from Health Canada, through the Canadian Partnership Against Cancer. We also acknowledge the Canadian Nuclear Safety Commission for reviewing the safety systems guideline.

## COPYRIGHT

This work is licensed under a Creative Commons Attribution 3.0 Unported License.
